# Ectonucleotidases CD39 and CD73 expression levels are independent and inverse predictors of survival in muscle‐invasive bladder cancer

**DOI:** 10.1002/2056-4538.70102

**Published:** 2026-06-23

**Authors:** Stephan Ledderose, Julia Schwenke, Lennert Eismann, Severin Rodler, Martina Rudelius, Carola Ledderose

**Affiliations:** ^1^ Institute of Pathology Ludwig Maximilian University Munich Munich Germany; ^2^ Department of Urology Ludwig Maximilian University Munich Munich Germany; ^3^ Department of Urology University Hospital of Schleswig‐Holstein, Campus Kiel Kiel Germany; ^4^ Department of Surgery University of California San Diego Health San Diego CA USA

**Keywords:** CD39, CD73, bladder cancer, purinergic signaling, biomarker, prognosis, tumor microenvironment

## Abstract

Bladder cancer is one of the leading causes of cancer‐related mortality worldwide. Long‐term survival is particularly poor in patients with muscle‐invasive bladder cancer (MIBC). Modulation of purinergic signaling through the ectonucleotidases CD39 and CD73 has emerged as a promising therapeutic strategy in cancer medicine. Altered expression patterns of these molecules have been linked to prognosis in various malignancies. In this study, we assessed the value of CD39 and CD73 expression as prognostic markers and potential therapeutic targets in MIBC. CD39 and CD73 expression was determined by immunohistochemistry using tissue microarrays from 180 patients with MIBC. Associations between tumoral and stromal expression and clinicopathological variables, including overall survival (OS), tumor‐specific survival (TSS) and disease‐free survival (DFS), were analyzed. Tumor cells did not express CD39. High stromal CD39 expression was significantly associated with a lower T category and UICC stage as well as prolonged median OS, TSS, and DFS. High CD73 expression by tumor cells was significantly associated with poorer OS and TSS. Stromal CD73 expression was not significantly correlated with survival outcomes. Our findings indicate distinct and compartment‐specific roles for CD39 and CD73 in MIBC. They suggest that high CD73 expression in tumor cells and low CD39 expression in stromal cells are negative prognostic indicators and potential therapeutic targets in MIBC.

## Introduction

Bladder cancer is one of the most common malignancies with a male to female ratio of 3:1 [1]. About 25% of newly diagnosed cases are classified as muscle‐invasive bladder cancer (MIBC), defined by tumor invasion into the detrusor muscle or beyond [2]. Despite advances in surgical and systemic therapies, MIBC remains associated with a poor prognosis [3]. Current standard of care consists of neoadjuvant chemotherapy followed by radical cystectomy with pelvic lymph node dissection [4]. However, up to half of the patients experience disease relapse within 5 years. Moreover, these comparatively aggressive treatment approaches are frequently associated with severe side effects and impaired quality of life [3, 5]. Improved prognostic biomarkers and novel therapeutic strategies are therefore urgently needed [6].

MIBC is characterized by a dynamic tumor microenvironment (TME) that profoundly influences cancer initiation, progression, and metastasis and has a strong impact on antitumoral immune surveillance [7, 8, 9]. Purinergic signaling has recently been recognized as a versatile modulator within the TME [10, 11]. Purine nucleotides and nucleosides, such as adenosine 5′‐triphosphate (ATP) and its metabolites adenosine diphosphate (ADP) and adenosine, act through a complex network of purinergic receptors, thereby influencing various cellular functions such as apoptosis, cell proliferation, cell migration, and differentiation [12, 13, 14, 15].

ATP is released actively or passively into the TME by stressed or dying cancer cells, immune cells, and stromal cells. Once released, it functions as an extracellular messenger molecule that activates cell surface‐bound purinergic P2 receptors, which comprise seven P2X (P2X1–P2X7) and eight P2Y (P2Y1, P2Y2, P2Y4, P2Y6, P2Y11–P2Y14) receptors [10, 14]. Several studies have shown that activation of P2X receptors by extracellular ATP affects tumor growth and progression across various cancer entities, including bladder cancer [10, 16, 17, 18]. Recently, we demonstrated that ATP has growth‐promoting effects on bladder cancer cells and that high expression levels of P2X1 and P2X7 receptors in MIBC correlate with poor outcome [18]. However, ATP can also promote anti‐tumoral immune responses through stimulation of P2X receptors on immune cells (reviewed in [19]). To add yet another layer of complexity, the ATP breakdown product adenosine modulates pro‐ and anti‐tumorigenic responses through four different adenosine receptors (A1, A2A, A2B, and A3) [20, 21, 22]. Consequently, the dominant effect of purinergic signaling – ranging from immunostimulatory, cancer cell growth‐inhibiting to immunosuppressive, growth‐promoting effects – is determined by the generation and turnover of purinergic ligands in the TME, the ratio of purine nucleotides to nucleosides, and the expression patterns of purinergic receptors on tumor, stromal, and immune cells [10, 11].

The stepwise hydrolysis of ATP to adenosine determines extracellular nucleotide concentrations and is mainly mediated by the membrane‐bound ectonucleotidases CD39 (also known as ectonucleoside triphosphate diphosphohydrolase 1, ENTDP1) and CD73 (ecto‐5′‐nucleotidase) [15]. CD39 catalyzes the conversion of ATP and ADP to adenosine monophosphate (AMP), while CD73 hydrolyzes AMP to adenosine [20]. Elevated extracellular adenosine levels promote T cell suppression and inhibit natural killer cell maturation and cytotoxic function, thereby contributing to tumor immune evasion and progression [21, 23, 24, 25, 26, 27].

By regulating the balance between ATP and adenosine in the TME, CD39, and CD73 can promote either pro‐ or anti‐tumorigenic conditions [20]. While high CD73 expression in tumor cells has been linked to poor clinical outcome, CD73 expression in tumor‐adjacent stromal cells is associated with improved prognosis in prostate and rectal cancer [28, 29, 30]. On the other hand, stromal CD39 expression correlates with favorable survival in lung cancer [31], but tumoral CD39 expression seems to predict poor survival in gastric and ovarian cancer [32, 33].

The expression patterns of CD39 and CD73 and their prognostic significance in MIBC have not been characterized yet [34]. A better understanding of these enzymes in MIBC may reveal novel mechanistic links between purinergic metabolism and clinical outcome, thereby aiding risk stratification and therapeutic decision‐making [35]. Moreover, deeper insights into this signaling axis may lead to the identification of new therapeutic targets [18, 36, 37].

In this study, we analyzed CD39 and CD73 expression patterns in 180 MIBC specimens. We found that stromal CD39 expression is significantly associated with improved long‐term survival, whereas tumoral CD73 expression is linked to a less favorable prognosis. These results suggest that compartment‐specific expression of CD39 and CD73 may serve as a prognostic biomarker and potential target for novel anti‐cancer therapies in MIBC.

## Materials and methods

### Patient cohort and sample details

The study was approved by the Medical Ethics Committee of Ludwig Maximilian University (LMU) Munich (approval number: 20‐179) and all procedures were conducted in accordance with the Declaration of Helsinki in its present form. Tumor samples from 180 patients who underwent radical cystectomy for MIBC at the Department of Urology (LMU Munich) between 2004 and 2014 were retrospectively evaluated. All surgical procedures were performed by experienced urologists using standardized surgical techniques. Histopathological assessment of all specimens was conducted by board‐certified pathologists of the Institute of Pathology (LMU Munich). Each case was systematically reviewed to confirm tumor histology, grade, and stage. Tumor staging and classification were standardized according to the AJCC/UICC TNM system (8th edition) and the WHO Classification of Urinary and Male Genital Tumors (5th edition) [38, 39].

Patients who had received neoadjuvant therapy or prior intravesical therapy with Bacillus Calmette‐Guerin or mitomycin were excluded. Additional exclusion criteria were incomplete tissue material or medical documentation, low‐grade tumors, and malignancies that did not meet the 2022 WHO histopathological definitions of urothelial carcinoma or its divergent differentiations and histomorphological subtypes. Follow‐up was performed in accordance with the European Association of Urology guidelines, either within the LMU Urology Department or by certified external urologists. Clinical and pathological parameters, including patient characteristics, overall survival (OS), tumor‐specific survival (TSS), and disease‐free survival (DFS), were recorded and correlated with CD39 and CD73 expression profiles.

### Tissue microarray construction

Protein expression of CD39 and CD73 was evaluated by immunohistochemistry using tissue microarrays (TMAs) generated from formalin‐fixed paraffin‐embedded (FFPE) tumor specimens from 180 patients with MIBC. From each sample, three 1‐mm cores representing distinct tumor regions were extracted and assembled into new TMA blocks to address intratumoral heterogeneity. Sections of 4 μm thickness were prepared from these arrays and subjected to immunohistochemical analysis.

### Immunohistochemistry

For CD39 staining, antigen retrieval was performed by heat treatment using Q Buffer EDTA at pH 8.0 (Quartett, Berlin, Germany; BU‐002‐0120). The slides were then incubated at room temperature for 60 min with a rabbit monoclonal anti‐human CD39 antibody (clone EPR26473‐58; 1:1000; Abcam, Cambridge, UK; ab300065). Bound antibodies were detected with the ImmPRESS Anti‐Rabbit IgG Polymer Kit (Vector Laboratories, Newark, CA, USA; MP‐7401).

For CD73 staining, heat‐induced epitope retrieval was carried out with Novocastra Epitope Retrieval Solution (pH 8.0; Leica Biosystems, Wetzlar, Germany; RE7116). The slides were then incubated for 60 min at room temperature with a rabbit monoclonal anti‐human CD73 antibody (clone D7F9A, 1:180; Cell Signaling Technology, Leiden, Netherlands; #13160). Bound antibodies were visualized using the MACH 3 Rabbit HRP Polymer Detection Kit (BioCare, Pacheco, CA, USA; M3R531).

All immunoreactions were developed using DAB+ as the chromogen (Agilent Technologies, Santa Clara, CA, USA; K3468) followed by counterstaining with hematoxylin (Vector Laboratories; H‐3401), dehydration, and mounting.

The antibodies used to detect CD39 and CD73 were validated by the manufacturers for use in Western blot and immunohistochemistry on FFPE tissues and have been widely used in previously published studies [40, 41].

### Semiquantitative analysis of ectonucleotidase expression

Immunostaining for CD39 and CD73 in both tumor cells and the surrounding stromal tissue (fibroblasts, endothelial cells, immune cells and smooth muscle cells) was evaluated separately using the histochemical scoring (*H*‐score) method as previously described [18]. This scoring approach incorporates two parameters: the staining intensity and the proportion of positively stained cells at each intensity level. Staining intensity was graded on a scale from 0 to 3 (0 = no, 1 = weak, 2 = moderate, 3 = strong staining). For each intensity level, the corresponding intensity score was multiplied by the percentage of positively stained cells. The *H*‐score was calculated by summing these values across all intensity levels. To account for tumor heterogeneity, the *H*‐scores of three tumor cores per sample were averaged to obtain the final *H*‐score. The median *H*‐score served as the threshold to define high nucleotidase expression (tumoral CD39: *H*‐score >0; stroma CD39: *H*‐score >60; tumoral CD73: *H*‐score >0; stromal CD73: *H*‐score >5). *H*‐scores were assigned by three pathologists (SL, JS, MR) with extensive experience in the analysis and interpretation of immunohistochemical stains. Joint evaluation and consensus finding were conducted in challenging cases to minimize interobserver variability.

### Statistical analysis

Statistical analyses were done with SigmaPlot version 12.5 (Systat Software Inc., San Jose, CA, USA). OS was defined as the interval from the date of primary surgery to death from any cause. TSS comprises the time from surgery to death attributable to bladder cancer. Deaths from other causes and patients alive at the last follow‐up were treated as censored observations. DFS equals the time from surgery to the time of disease recurrence. Patients without evidence of recurrence or metastasis at the end of follow‐up or who died without documented relapse were censored. Survival curves were created by the Kaplan–Meier method and compared with the log‐rank test.

Correlations between ectonucleotidase expression and clinicopathological or demographic variables were first broadly analyzed with the Spearman rank correlation test and a heatmap was used to visualize the results. In addition, associations between high or low CD73 or CD39 expression and the parameters age, sex, pathological T category (pT), nodal status (pN), presence of distant metastasis (M), lymphovascular invasion (L), vascular invasion (V), perineural invasion (Pn), resection margin status (R), Union for International Cancer Control (UICC) stage and receipt of adjuvant therapy were analyzed using the chi‐square test for categorical variables and the unpaired *t*‐test for continuous variables.

To evaluate the prognostic significance of CD39 and CD73 expression levels (categorized as low versus high), multivariate Cox proportional hazards regression analyses were performed. Models were adjusted for the confounders age, pT category, and UICC stage. Statistical significance was assumed for *p* < 0.05.

## Results

### Demographics and clinicopathological characteristics

A total of 180 MIBC patients were enrolled in the study. Of those, 62 patients (34.4%) underwent adjuvant chemotherapy, and 43 patients (23.9%) received adjuvant radiotherapy. Consistent with previous reports, most patients were male (73.3%) [42]. The average age at the time of surgery was 67.4 years (±9.7). The mean follow‐up period was 3 years with 150 patients (83.3%) dying within this period. Clinicopathological characteristics of the study population, including pT and pN categories, UICC stage and M, R, L, V, and Pn status are shown in Table 1.

**Table 1:** Associations of demographic and clinicopathological characteristics with CD73 and CD39 expression

		CD73 tumor			CD39 stroma			CD73 stroma		
	Study population, *n* = 180	Low, *n* = 133	High, *n* = 47	*p*	Low, *n* = 90	High, *n* = 90	*p*	Low, *n* = 106	High, *n* = 74	*p*
Age at diagnosis (years)										
Mean (SD)	67.4 (9.7)	68.0 (10.0)	65.6 (8.6)	0.15*	69.3 (9.7)	65.4 (9.4)	**0.01** *	66.1 (10.4)	69.1 (8.4)	**0.04** *

	Study population, *n* (%)	CD73 tumor			CD39 stroma			CD73 stroma		
		Low, *n* (%)	High, *n* (%)	*p*	Low, *n* (%)	High, *n* (%)	*p*	Low, *n* (%)	High, *n* (%)	*p*
T stage				0.13^†^			**0.01** ^†^			**0.03** ^†^
T2	34 (18.9)	26 (19.5)	8 (17.0)		10 (11.1)	24 (26.7)		20 (18.9)	14 (18.9)	
T3	104 (57.8)	81 (60.9)	23 (48.9)		53 (58.9)	51 (56.7)		54 (50.9)	50 (67.6)	
T4	42 (23.3)	26 (19.5)	16 (34.0)		27 (30.0)	15 (16.7)		32 (30.2)	10 (13.5)	
Lymph node status^‡^				0.77^†^			0.51^†^			0.49^†^
pN0	101 (61.2)	76 (62.3)	25 (58.1)		47 (58.0)	54 (64.3)		58 (58.6)	43 (65.2)	
pN+	64 (38.8)	46 (37.7)	18 (41.9)		34 (42.0)	30 (35.7)		41 (41.4)	23 (34.8)	
UICC stage				0.61^†^			**<0.001** ^†^			0.12^†^
2	28 (15.6)	21 (15.8)	7 (14.9)		5 (5.6)	23 (25.6)		17 (16.0)	11 (14.9)	
3	67 (37.2)	52 (39.1)	15 (31.9)		36 (40.0)	31 (34.4)		33 (31.1)	34 (45.9)	
4	85 (47.2)	60 (45.1)	25 (53.2)		49 (54.4)	36 (40.0)		56 (52.8)	29 (39.2)	
Vascular invasion				0.58^†^			0.84^†^			1.00^†^
V0	152 (84.4)	114 (85.7)	38 (80.9)		75 (83.3)	77 (85.6)		90 (84.9)	62 (83.8)	
V1	28 (15.6)	19 (14.3)	9 (19.1)		15 (16.7)	13 (14.4)		16 (15.1)	12 (16.2)	
Lymphovascular invasion				0.21^†^			0.37^†^			0.20^†^
L0	81 (45.0)	64 (48.1)	17 (36.2)		37 (41.1)	44 (48.9)		43 (40.6)	38 (51.4)	
L1	99 (55.0)	69 (51.9)	30 (63.8)		53 (58.9)	46 (51.1)		63 (59.4)	36 (48.6)	
Perineural invasion				0.28^†^			1.00^†^			0.45^†^
Pn0	133 (73.9)	95 (71.4)	38 (80.9)		67 (74.4)	66 (73.3)		81 (76.4)	52 (70.3)	
Pn1	47 (26.1)	38 (28.6)	9 (19.1)		23 (25.6)	24 (26.7)		25 (23.6)	22 (29.7)	
Distant metastasis				0.45^†^			0.66^†^			0.38^†^
M0	157 (87.2)	118 (88.7)	39 (83.0)		77 (85.6)	80 (88.9)		90 (84.9)	67 (90.5)	
M1	23 (12.8)	15 (11.3)	8 (17.0)		13 (14.4)	10 (11.1)		16 (15.1)	7 (9.5)	
Resection margin				0.37^†^			0.42^†^			0.56^†^
R0	151 (83.9)	114 (85.7)	37 (78.7)		73 (81.1)	78 (86.7)		87 (82.1)	64 (86.5)	
R1	29 (16.1)	19 (14.3)	10 (21.3)		17 (18.9)	12 (13.3)		19 (17.9)	10 (13.5)	
Adjuvant chemotherapy				0.91^†^			0.27^†^			0.34^†^
Yes	62 (34.4)	46 (34.6)	16 (34.0)		35 (38.9)	27 (30.0)		40 (37.7)	22 (29.7)	
No	118 (65.6)	87 (65.4)	31 (66.0)		55 (61.1)	63 (70.0)		66 (62.3)	52 (70.3)	
Adjuvant radiotherapy				0.28^†^			0.48^†^			0.95^†^
Yes	43 (23.9)	35 (26.3)	8 (17.0)		24 (26.7)	19 (21.1)		25 (23.6)	18 (24.3)	
No	137 (76.1)	98 (73.7)	39 (83.0)		66 (73.3)	71 (78.9)		81 (76.4)	56 (75.7)	

Bold font in results indicates statistical significance.

SD, standard deviation.

* *t*‐test.

^†^ Chi‐square test.

^‡^ Lymph node status unknown in 15 patients.

### CD73 and CD39 expression patterns are highly variable in MIBC

**Figure 1:**
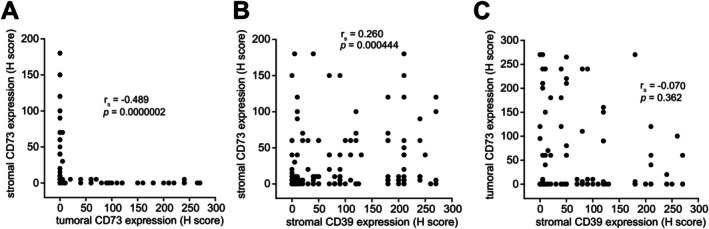
Weak correlation of compartmentalized CD73 and CD39 expression in muscle‐invasive bladder cancer (MIBC). Expression levels of CD73 and CD39 in stroma and tumor cells of MIBC samples (*n* = 180) were analyzed by immunohistochemistry and *H*‐scores were assigned. Associations between tumoral and stromal CD73 expression (A), stromal CD73 and CD39 expression (B), and tumoral CD73 and stromal CD39 expression (C) were tested for significance using the Spearman rank correlation test (*r*_s_: Spearman correlation coefficient). Tumoral CD39 expression was not detected in any sample.

CD73 and CD39 expression in MIBC varied considerably between patients in both intensity and localization to tumor or stroma cells. Comparing absolute *H*‐scores, we found an inverse relationship between tumoral and stromal CD73 expression. Stromal CD73 expression was highest in tumor samples lacking or showing only minimal tumoral CD73 expression, whereas strong tumoral CD73 positivity was associated with weak stromal staining. Accordingly, a moderate negative correlation between tumoral and stromal CD73 expression was identified (*r*_s_ = −0.489, *p* < 0.001; Figure 1A), indicating that CD73 expression preferentially localized either to the tumor cell compartment or to the surrounding stroma, but not to both simultaneously.

CD39 expression was exclusively detected in stromal cells. Interestingly, stromal CD39 expression showed a weak but statistically significant positive correlation with stromal CD73 expression (*r*_s_ = 0.26, *p* < 0.001; Figure 1B). There was no association between stromal CD39 expression and CD73 expression in tumor cells (Figure 1C).

**Figure 2:**
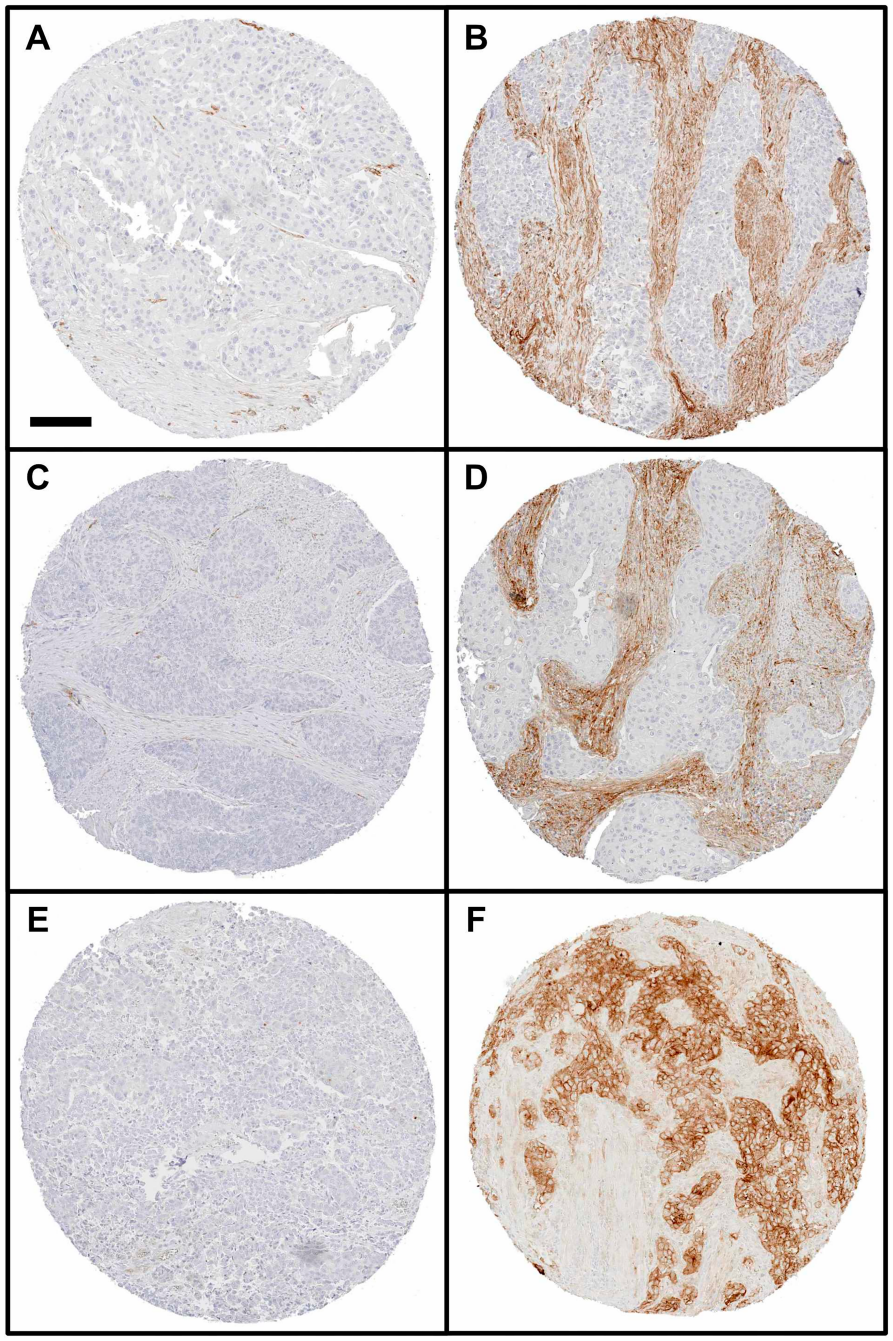
Compartment‐dependent CD39 and CD73 expression in muscle‐invasive bladder cancer (MIBC). (A and B) Representative photomicrographs show TMA samples of MIBC with low (A) and high (B) stromal CD39 expression. (C and D) TMA samples of MIBC with low (D) and high (E) stromal CD73 expression are shown. (E and F) Low and high tumoral CD73 expression patterns in MIBC are shown in (E) and (F), respectively. There were no MIBC samples with CD39 expression in tumor cells. Scale bar equals 150 μm.

To assess the prognostic value of compartment‐specific CD73 and CD39 expression in MIBC, we defined cut‐off *H*‐scores and categorized samples as having low or high enzyme expression in stromal or tumor cells. High CD73 expression in tumor cells was observed in 47 cases (26.1%), while elevated stromal CD73 expression was noted in 74 cases (41.1%). Additionally, half of the cases (*n* = 90, 50%) demonstrated high stromal CD39 expression. Interestingly, CD39 expression was not detected in tumor cells. Representative microphotographs of CD39 and CD73 expression patterns are shown in Figure 2.

### Ectonucleotidase expression and OS

Since we had found clear differences in the expression patterns of CD73 and CD39, we hypothesized that these enzymes may affect tumor progression and survival in MIBC patients. To test this assumption, we stratified our study group based on expression levels and compared OS, TSS, and DFS between the subgroups.

**Figure 3:**
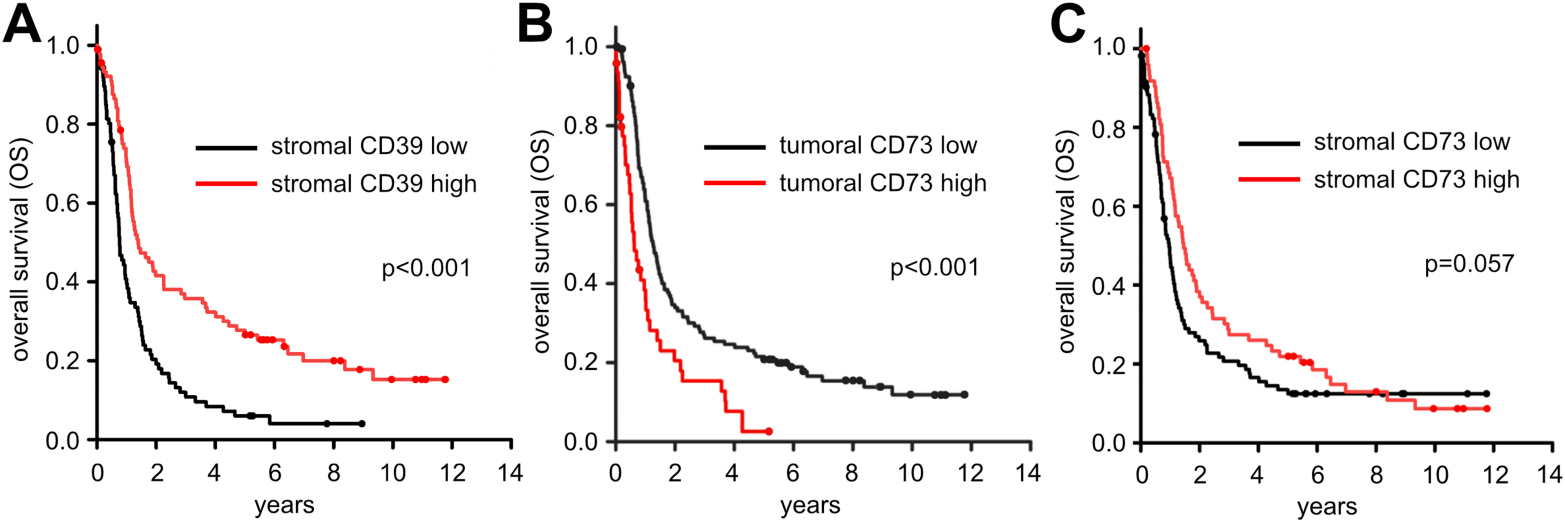
Prognostic significance of CD39 and CD73 expression for overall survival (OS) in muscle‐invasive bladder cancer (MIBC). (A) MIBC patients with high stromal CD39 expression showed a significant improved OS compared to patients with low stromal CD39 expression (A, *p* < 0.001). (B and C) While patients with low tumoral CD73 expression had a better OS than patients with high tumoral CD73 expression (B, *p* < 0.001), stromal CD73 expression had no statistically significant influence on OS (C, *p* = 0.057).

Median OS of all patients after initial surgery was 18.4 months [95% confidence interval (CI): 15.5–21.3]. Patients with a high stromal CD39 immunoreactivity showed a significant increase in OS (22.8 months; 95% CI: 13.9–31.7) as compared to patients with a low stromal CD39 score (12.7 months; 95% CI: 9.9–16.4; *p* < 0.001; Figure 3A). OS in patients with low CD73 expression in tumor cells was 20.9 months (95% CI: 17.1–24.7) and significantly higher than in patients with high tumoral CD73 expression (10.3 months; 95% CI: 5.8–14.8; *p* < 0.001; Figure 3B). Differences in OS between patients with high stromal CD73 expression (24.0 months; 95% CI: 18.3–29.7) compared to those with low stromal CD73 expression (15.8 months, 95% CI: 13.0–18.6) were not statistically significant (*p* = 0.057; Figure 3C).

### Ectonucleotidase expression and TSS

**Figure 4:**
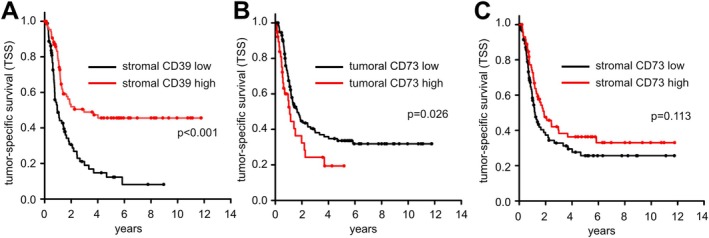
Prognostic significance of CD39 and CD73 expression for tumor‐specific survival (TSS) in muscle‐invasive bladder cancer (MIBC). (A) High stromal CD39 expression was significantly associated with an improved TSS of MIBC patients compared to low stromal CD39 expression (A, *p* < 0.001). (B and C) While patients with low tumoral CD73 expression had a significantly improved TSS compared to patients with high tumoral CD73 expression (B, *p* = 0.026), stromal CD73 expression had no statistically significant influence on TSS (C, *p* = 0.113).

MIBC‐associated death occurred in 100 patients (55.6%) and median TSS was 23.4 months (95% CI: 15.7–31.1). Patients with a high stromal CD39 score showed a significant increase in TSS (48.7 months, 95% CI: not reached) as compared to patients with a low stromal CD39 score (15.8 months; 95% CI: 10.2–24.4; *p* < 0.001; Figure 4A). TSS in patients with a high CD73 expression in tumor cells (17.8 months; 95% CI: 13.1–22.5) was significantly lower than in patients with a low CD73 tumoral expression (30.4 months; 95% CI: 18.5–35.5; *p* = 0.026; Figure 4B). Consistent with the lack of an effect of stromal CD73 on OS (Figure 3C), patients with high stromal CD73 expression showed no significant difference in TSS (30.9 months; 95% CI: 16.5–45.3) compared to patients with low stromal CD73 expression (19.7 months; 95% CI: 14.9–21.4; *p* = 0.113; Figure 4C).

### Ectonucleotidase expression and DFS

**Figure 5:**
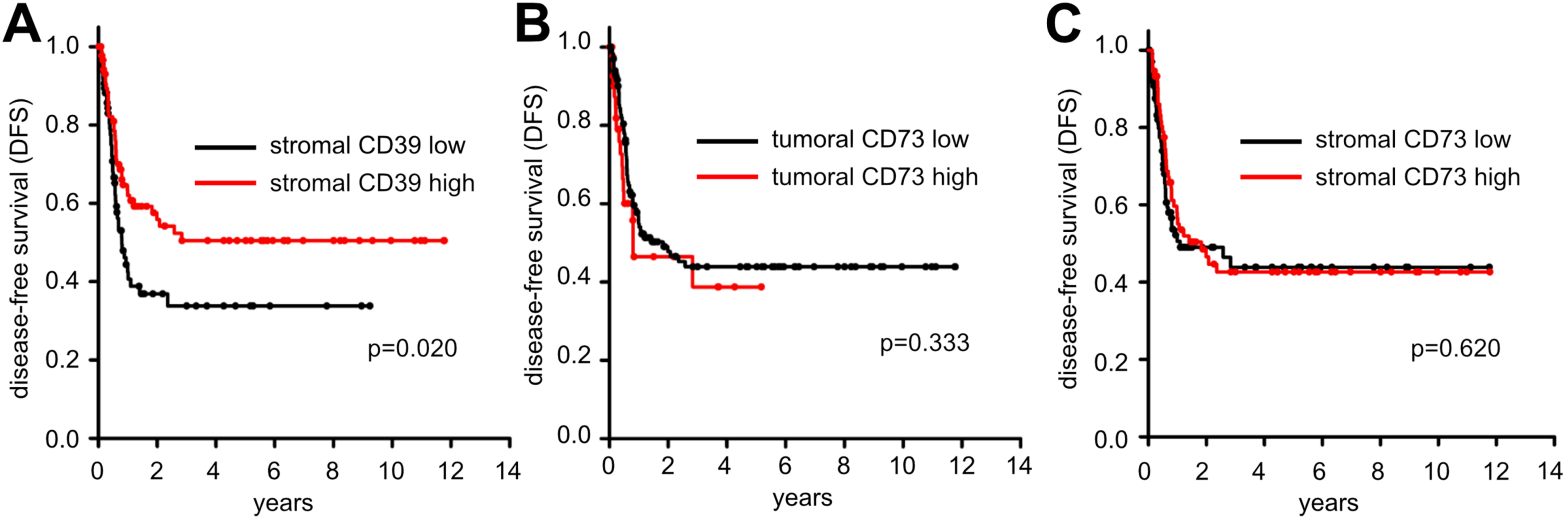
Prognostic significance of CD39 and CD73 expression for disease‐free survival (DFS) in muscle‐invasive bladder cancer (MIBC). (A) MIBC patients with high stromal CD39 expression showed a significant improvement in DFS compared to patients with low stromal CD39 expression (A, *p* < 0.020). (B and C) Tumoral and stromal CD73 expression had no statistically significant influence on DFS (B, *p* = 0.333; C, *p* = 0.620).

Median DFS of all patients was 23.0 months (95% CI: 4.0–42.0). DFS was significantly increased in patients with high stromal CD39 expression compared to patients with low stromal CD39 expression. While patients with low stromal CD39 scores had a median DFS of 13.0 months (95% CI: 8.3–17.8), the median DFS for patients with high stromal CD39 expression was not reached as more than 50% of the patients were still relapse‐free at the time of analysis, indicating superior long‐term outcomes for this cohort (*p* = 0.020; Figure 5A). The differences in DFS between the subgroups with low and high expression of CD73 in tumoral or stromal cells were not statistically significant (*p* = 0.333 and *p* = 0.620, respectively; Figure 5B,C).

### Associations between CD73 or CD39 expression and demographic and clinicopathological parameters

The results of the survival analyses above suggested that CD73 and CD39 expression levels might indeed predict outcome in MIBC patients. To further evaluate their potential as independent predictors, we first created a correlation matrix of tumoral CD73 and stromal CD73 and CD39 *H*‐scores and well‐established clinical (age, sex, adjuvant therapy) and histopathological (pT and pN categories, UICC stage and M, R, L, V, and Pn status) prognostic parameters (supplementary material, Figure S1). As expected, there were strong positive correlations between the UICC stage and parameters such as pT, pN, M, L, and R status. Associations between ectonucleotidase expression levels and histopathological variables were overall low.

Next, we used the stratified expression data to analyze whether there were differences between the high and low expression groups with regard to the above‐mentioned clinical and histopathological parameters. No significant associations were identified between these factors and CD73 expression in tumor cells. However, high stromal CD73 expression was significantly linked to a higher age (*p* = 0.04) and lower pT category (*p* = 0.03). Similarly, high stromal CD39 expression was significantly associated with lower age (*p* = 0.01), lower pT category (*p* = 0.01), and lower UICC stage (*p* < 0.001). The results of these analyses are summarized in Table 1.

### Multivariate analyses

To test whether CD73 and CD39 expression could serve as independent prognostic factors, we calculated multivariate survival models for OS, TSS, and DFS with adjustments for age, pT category, and UICC stage. Elevated stromal CD39 expression was identified as an independent factor associated with improved OS [hazard ratio (HR) = 0.70; 95% CI: 0.49–0.99; *p* = 0.04] and TSS (HR = 0.57; 95% CI: 0.37–0.87; *p* = 0.01) in patients with MIBC. High CD73 expression in tumor cells was independently linked to poorer OS (HR = 2.12; 95% CI: 1.37–3.30; *p* < 0.001). In agreement with the survival analyses (Figures 3, 4, 5), stromal CD73 levels were not associated with survival outcomes. The results of the multivariate analysis are summarized in Table 2.

**Table 2:** Multivariate analysis of potential independent prognostic survival factors in muscle‐invasive bladder cancer patients

	*n* (%)	OS			TSS			DFS		
		HR	95% CI	*p*	HR	95% CI	*p*	HR	95% CI	*p*
Age										
<70 years	107 (59.4)	Reference								
>70 years	73 (40.6)	1.39	0.97–1.98	0.07	0.93	0.59–1.44	0.73	1.29	0.81–2.05	0.27
T stage										
T2–T3	138 (76.7)	Reference								
T4	42 (23.3)	1.26	0.87–1.85	0.22	1.58	1.02–2.45	**0.04**	1.52	0.94–2.47	0.09
UICC stage										
2	28 (15.6)	Reference								
3–4	152 (84.4)	6.02	3.08–11.76	**<0.001**	8.62	3.08–24.40	**<0.001**	11.96	2.87–50.0	**<0.001**
CD39 stroma										
Low	90 (50)	Reference								
High	90 (50)	0.70	0.49–0.99	**0.04**	0.57	0.37–0.87	**0.01**	0.88	0.56–1.40	0.59
CD73 stroma										
Low	106 (58.9)	Reference								
High	74 (41.1)	0.86	0.58–1.27	0.44	0.90	0.56–1.44	0.66	0.93	0.56–1.55	0.79
CD73 tumor										
Low	133 (73.9)	Reference								
High	47 (26.1)	2.12	1.37–3.30	**<0.001**	1.44	0.83–2.49	0.19	1.43	0.78–2.62	0.25

Bold font in results indicates statistical significance.

CI, confidence interval; DFS, disease‐free survival; HR, hazard ratio; OS, overall survival; TSS, tumor‐specific survival.

## Discussion

In this study, we found that CD39 and CD73 expression has a distinct and compartment‐specific impact on long‐term survival in MIBC patients undergoing radical cystectomy. While CD39 was not expressed in tumor cells, high stromal CD39 expression was associated with improved survival. Conversely, elevated CD73 expression in tumor cells correlated with poor prognosis, whereas stromal CD73 expression was not significantly associated with outcome. These findings suggest that the composition of purinergic ligands in different compartments of the TME significantly affects tumor growth and anti‐tumor responses.

ATP and adenosine are the primary ligands of the 19 purinergic receptors expressed by mammalian cells [14]. Depending on ligand concentrations and receptor expression patterns, purinergic signaling can either promote or inhibit tumor growth and antitumor immunity [10, 35]. ATP was shown to facilitate tumor progression in several cancers, including bladder cancer [10, 17, 18, 43]. Recently, we demonstrated that ATP promotes bladder cancer cell proliferation *via* activation of P2X1 and P2X7 receptors, most likely through calcium‐dependent pathways that influence cellular energy metabolism [17, 18, 44, 45]. We also found that high‐grade urothelial cancer cells maintain higher ATP concentrations in their surroundings than low‐grade urothelial cancer cells. Consistently, high P2X1/P2X7 expression in MIBC correlates with poor outcome [18].

CD39 and CD73 are the two major ectonucleotidases that regulate extracellular concentrations of ATP and adenosine [15]. CD39 catalyzes the hydrolysis of ATP to ADP and AMP, which is then converted to adenosine by CD73 [46]. The CD39/CD73 axis has been associated with adenosine‐mediated immunosuppression, impaired antitumor immunity, and tumor progression in several cancers [20, 24].

In our MIBC cohort, stromal CD39 expression was associated with improved survival. One possible explanation for this apparent anti‐tumoral effect of CD39 in MIBC patients is the reduction of peritumoral ATP levels by CD39‐expressing stromal cells, leading to a disruption of ATP‐driven pro‐tumorigenic P2X signaling and ultimately a better prognosis [18]. This effect may outweigh the immunosuppressive effect of adenosine production and is consistent with recent findings in non‐small cell lung cancer, where stromal CD39 expression was also associated with improved survival [31]. Contrary to MIBC, CD39 is expressed by non‐invasive bladder cancer cells, which are much more capable of hydrolyzing extracellular ATP than high‐grade bladder cancer cells [34, 47], suggesting that high pericellular ATP concentrations are needed to develop muscle‐invasive potential.

Stromal CD39 expression may also reflect the presence of immune cell populations with antitumorigenic properties. CD39 is expressed by various immune cells, including regulatory T cells, activated T cells, macrophages, and dendritic cells [48, 49, 50]. While CD39 expression contributes to immunosuppressive regulatory T cell function [51], its expression on other immune cells has been associated with enhanced immunosurveillance and improved outcomes in several malignancies [48, 49, 52, 53, 54, 55]. Because this study aimed to find an easy‐to‐determine prognostic biomarker, we did not further differentiate stromal cell populations using more complex methods such as multiplex immunohistochemistry. However, future studies should address detailed cellular CD39 expression profiles within the MIBC microenvironment.

CD73 catalyzes the final, rate‐limiting step of adenosine generation [15]. Adenosine exerts mainly suppressive effects on immune cells [49, 56] and promotes angiogenesis and tumor survival under hypoxic conditions [20, 57, 58]. Consequently, tumor cell‐associated CD73 expression has been linked to immune evasion, therapy resistance, metastasis, and poor survival in multiple cancers, including colorectal carcinoma and pancreatic carcinoma [59, 60, 61, 62, 63, 64]. Consistent with these reports, tumoral CD73 expression was associated with poor prognosis in our MIBC cohort. Interestingly, there was a moderate inverse relationship between tumoral and stromal CD73 expression, but stromal CD73 expression did not significantly affect outcome. This may reflect context‐dependent effects of CD73 influenced by the composition of the TME [28, 29, 34, 37, 65, 66]. In MIBC, stromal CD73 alone may not be sufficient to generate adenosine concentrations high enough to exert a dominant biological effect, or its activity may be counterbalanced by other enzymatic or immunoregulatory mechanisms.

The opposite effect of stromal CD39 and tumoral CD73 expression on survival underlines the complex role of purinergic signaling in MIBC. Our data suggest that stromal CD39 limits ATP‐driven tumor progression, while tumoral CD73 expression may facilitate immune evasion by generating immunosuppressive adenosine. In support of these findings, *in silico* survival analyses of the cancer genome atlas (TCGA) bladder cancer datasets provided by the Human Protein Atlas (www.proteinatlas.org) showed that higher CD39 expression and lower CD73 expression are associated with improved outcome [67].

These findings have important translational implications. CD39 and CD73 can be easily assessed by immunohistochemistry on FFPE tissue samples and are likely well suited for inclusion in routine diagnostic panels. Our results suggest that compartment‐specific expression patterns of CD39 and CD73 may serve as adjunct prognostic biomarkers in MIBC patients, supporting risk stratification and individualized treatment decisions. For example, patients with high tumoral CD73 or low stromal CD39 expression may benefit from intensified surveillance and more aggressive treatment approaches.

Therapeutic strategies targeting purinergic signaling, including inhibitors of CD73, CD39, and adenosine receptors, are currently being investigated [36, 37, 68, 69]. Our results suggest that MIBC patients with high tumoral CD73 expression may benefit from CD73‐directed therapies or inhibition of adenosine‐signaling as this could restore antitumor immunity. In contrast, inhibition of CD39 appears less promising, given its absence in MIBC cancer cells and its association with favorable outcomes when expressed in the stromal compartment. These considerations highlight the need for a detailed understanding of spatial expression patterns of CD39 and CD73.

Our study has some limitations. Although immunohistochemistry is widely available and well established in routine clinical practice, it provides no information about enzymatic activity and allows only limited assignment to specific cell types. Furthermore, although the *H*‐score is widely used for semiquantitative biomarker assessment and is broadly applied in translational research and selected diagnostic applications [70, 71], prospective multi‐center studies are needed to establish reproducible cut‐off values and interobserver reproducibility.

Another limitation is the use of TMAs, which may not fully capture intratumoral heterogeneity. To reduce sampling bias, we used multiple representative cores per tumor [72]. Approaches using whole‐slide sections and spatially resolved techniques may provide more comprehensive analyses of compartment‐specific CD39 and CD73 expression. Finally, the use of a single antibody per target is a methodological limitation and off‐target binding cannot be fully excluded. Future studies may validate and expand on our findings using complementary methods, including functional assays, spatial transcriptomics, and multiplex immunophenotyping. Given the growing clinical relevance of immunotherapy in MIBC, understanding interactions between purinergic signaling and immune checkpoint pathways will be particularly important. For example, early evidence suggests that adenosinergic mechanisms can impair the efficacy of checkpoint inhibitor therapy and that co‐targeting of purinergic signaling pathways may enhance anti‐tumor immune responses [73].

In conclusion, our study provides novel evidence that CD39 and CD73 are promising prognostic biomarkers and potential therapeutic targets in patients with MIBC. Prospective validation in independent patient cohorts and clinical trials will be necessary prior to implementation in routine practice.

## Author contributions statement

SL and CL conceived of the study. SL, JS, LE and SR collected the data. SL, JS and MR evaluated TMAs. SL analyzed the data with help of all authors. CL provided supervisory support. SL wrote the manuscript. CL, JS, LE, SR and MR reviewed the manuscript. All authors discussed the results and contributed to and approved of the final version of the manuscript.

## Supporting information


**Figure S1.** Clinicopathological correlations: compartment‐dependent CD39 and CD73 expression

## Data Availability

The data that support the findings of this study are available in this article.
