# Supplementary material for: Ectonucleotidases CD39 and CD73 expression levels are independent and inverse predictors of survival in muscle‐invasive bladder cancer
Source: J Pathol Clin Res. 2026 Jun 23;12(4):e70102. doi: 10.1002/2056-4538.70102 (PMC13288156; doi:10.1002/2056-4538.70102)
Supplement: Supplementary file 1 — Figure S1. Clinicopathological correlations: compartment‐dependent CD39 and CD73 expression [file CJP2-12-e70102-s001.pdf]

**Ectonucleotidases CD39 and CD73 expression levels are independent and inverse predictors of survival in muscle-invasive bladder cancer**

S Ledderose *et al*. *J Pathol Clin Res* https://doi.org/10.1002/2056-4538.70102

**Supplementary Figure S1**

| | CD73 Tumor H-Score | CD39 Stroma H-Score | CD73 Stroma H-Score | Age | pT | M | UICC | V | L | Pn | R | Adjuvant CT | Adjuvant RT | pN |
|---|---|---|---|---|---|---|---|---|---|---|---|---|---|---|
| **CD73 Tumor H-Score** | | **0.042** | **0.000** | 0.173 | 0.068 | 0.367 | **0.029** | 0.551 | 0.236 | 0.930 | 0.054 | 0.656 | 0.736 | 0.442 |
| **CD39 Stroma H-Score** | **0.042** | | **0.024** | **0.043** | 0.077 | **0.002** | 0.097 | 0.119 | 0.178 | 0.831 | 0.644 | 0.013 | 0.172 | 0.280 |
| **CD73 Stroma H-Score** | **0.000** | **0.024** | | 0.846 | 0.684 | 0.080 | 0.059 | 0.099 | 0.203 | 0.294 | 0.245 | 0.589 | 0,159 | 0.433 |
| **Age** | 0.173 | **0.043** | 0.846 | | 0.785 | 0.090 | 0.604 | 0.646 | 0.578 | 0.827 | 0.573 | **0.001** | 0,182 | 0.985 |
| **pT** | 0.068 | 0.077 | 0.684 | 0.785 | | **0.016** | **0.000** | 0.385 | **0.001** | **0.039** | **0.000** | **0.000** | **0.014** | 0.688 |
| **M** | 0.367 | **0.002** | 0.080 | 0.090 | **0.016** | | **0.000** | 0.796 | **0.004** | 0.313 | 0.435 | **0.017** | 0.793 | **0.036** |
| **UICC** | **0.029** | 0.097 | 0.059 | 0.604 | **0.000** | **0.000** | | 0.244 | **0.000** | 0.234 | **0.000** | **0.000** | **0.006** | **0.000** |
| **V** | 0.551 | 0.119 | 0.099 | 0.646 | 0.385 | 0.796 | 0.244 | | 0.805 | 0.885 | 0.166 | 0.879 | 0.882 | 0.637 |
| **L** | 0.236 | 0.178 | 0.203 | 0.578 | **0.001** | **0.004** | **0.000** | 0.805 | | 0.466 | 0.014 | **0.000** | 0.061 | **0.000** |
| **Pn** | 0.930 | 0.831 | 0.294 | 0.827 | **0.039** | 0.313 | 0.234 | 0.885 | 0.466 | | 0.793 | 0.947 | **0.022** | 0.633 |
| **R** | 0.054 | 0.644 | 0.245 | 0.573 | **0.000** | 0.435 | **0.000** | 0.166 | **0.014** | 0.793 | | 0.996 | 0.053 | **0.031** |
| **Adjuvant CT** | 0.656 | **0.013** | 0.589 | **0.001** | **0.000** | **0.017** | **0.000** | 0.879 | **0.000** | 0.947 | 0.996 | | 0.243 | **0.015** |
| **Adjuvant RT** | 0.736 | 0.172 | 0.159 | 0.182 | **0.014** | 0.793 | **0.006** | 0.882 | 0.061 | **0.022** | 0.053 | 0.243 | | 0.096 |
| **pN** | 0.442 | 0.280 | 0.433 | 0.985 | 0.688 | **0.036** | **0.000** | 0.637 | **0.000** | 0.633 | **0.031** | **0.015** | 0.096 | |

Spearman correlation coefficient

- 0,25 0,0 1,0

**Figure S1.** Correlations between compartment-dependent CD39 and CD73 expression and clinicopathological parameters. The heatmap depicts color-coded Spearman correlation coefficients and corresponding *p* values. Bold print indicates statistical significance ($p < 0.05$).
